# Investigating the cardiac pathology of SCO2‐mediated hypertrophic cardiomyopathy using patients induced pluripotent stem cell–derived cardiomyocytes

**DOI:** 10.1111/jcmm.13392

**Published:** 2017-11-28

**Authors:** Tova Hallas, Binyamin Eisen, Yuval Shemer, Ronen Ben Jehuda, Lucy N. Mekies, Shulamit Naor, Revital Schick, Sivan Eliyahu, Irina Reiter, Eugene Vlodavsky, Yeshayahu (Shai) Katz, Katrin Õunap, Avraham Lorber, Richard Rodenburg, Hanna Mandel, Mihaela Gherghiceanu, Ofer Binah

**Affiliations:** ^1^ Department of Physiology, Biophysics and Systems Biology Technion Haifa Israel; ^2^ The Rappaport Institute Technion Haifa Israel; ^3^ Rappaport Faculty of Medicine Technion Haifa Israel; ^4^ Department of Biotechnology Technion Haifa Israel; ^5^ Department of Pathology Rambam Health Care Campus Haifa Israel; ^6^ Department of Anesthesiology Rambam Health Care Campus Haifa Israel; ^7^ Department of Genetics United Laboratories Tartu University Hospital Tartu Estonia; ^8^ Department of Pediatrics Institute of Clinical Medicine University of Tartu Tartu Estonia; ^9^ Department of Pediatric Cardiology Rambam Health Care Campus Haifa Israel; ^10^ Radboud Center for Mitochondrial Disorders Translational Metabolic Laboratory Department of Pediatrics Radboud University Medical Center Nijmegen The Netherlands; ^11^ Metabolic Unit Department of Pediatrics Rambam Health Care Campus Haifa Israel; ^12^ ‘Victor Babes’ National Institute of Pathology Bucharest Romania

**Keywords:** SCO2 mutation, HCM, cardiomyocytes, iPSC, [Ca^2+^]_i_ transients and contractions, action potentials, arrhythmias

## Abstract

Mutations in SCO2 are among the most common causes of COX deficiency, resulting in reduced mitochondrial oxidative ATP production capacity, often leading to hypertrophic cardiomyopathy (HCM). To date, none of the recent pertaining reports provide deep understanding of the SCO2 disease pathophysiology. To investigate the cardiac pathology of the disease, we were the first to generate induced pluripotent stem cell (iPSC)‐derived cardiomyocytes (iPSC‐CMs) from SCO2‐mutated patients. For iPSC generation, we reprogrammed skin fibroblasts from two SCO2 patients and healthy controls. The first patient was a compound heterozygote to the common E140K mutation, and the second was homozygote for the less common G193S mutation. iPSC were differentiated into cardiomyocytes through embryoid body (EB) formation. To test the hypothesis that the SCO2 mutation is associated with mitochondrial abnormalities, and intracellular Ca^2+^‐overload resulting in functional derangements and arrhythmias, we investigated in SCO2‐mutated iPSC‐CMs (compared to control cardiomyocytes): (*i*) the ultrastructural changes; (*ii*) the inotropic responsiveness to β‐adrenergic stimulation, increased [Ca^2+^]_o_ and angiotensin‐II (AT‐II); and (*iii*) the Beat Rate Variability (BRV) characteristics. In support of the hypothesis, we found in the mutated iPSC‐CMs major ultrastructural abnormalities and markedly attenuated response to the inotropic interventions and caffeine, as well as delayed afterdepolarizations (DADs) and increased BRV, suggesting impaired SR Ca^2+^ handling due to attenuated SERCA activity caused by ATP shortage. Our novel results show that iPSC‐CMs are useful for investigating the pathophysiological mechanisms underlying the SCO2 mutation syndrome.

## Introduction

Derangements in the mitochondrial respiratory chain and specifically in cytochrome c oxidase (COX) are associated with deleterious effects in organs with high energy demands, such as the heart. Consequently, cardiac pathologies are a prevalent outcome of mitochondrial‐related disorders 1. The human SCO2 gene encodes a 266‐amino‐acid metallochaperone that participates in copper delivery to COX 2, and thus mutations in SCO2 are among the most common causes of COX deficiency 3. These disorders are characterized by encephalopathy and HCM, collectively leading to death in infancy or early childhood 4. Based on the COX deficiency caused by SCO2 mutations (resulting in ATP shortage), we hypothesized that the SCO2 mutation is associated with mitochondrial abnormalities, and intracellular Ca^2+^‐overload resulting in functional derangements and arrhythmias. To test this hypothesis, we investigated in SCO2‐mutated iPSC‐CMs generated from two babies (who died at 3–4 months) carrying different SCO2 mutations: (*i*) the ultrastructural changes; (*ii*) the inotropic responsiveness to β‐adrenergic stimulation, increased [Ca^2+^]_o_ and AT‐II; and (*iii*) the Beat Rate Variability (BRV) characteristics. In support of our hypothesis, the diseased cardiomyocytes demonstrated abnormal mitochondrial ultrastructure, functional derangements including arrhythmias, suggesting disturbed intracellular Ca^2+^ homoeostasis, likely due to ATP deficiency.

## Materials and methods

### iPSC generation and characterization

Dermal biopsies were obtained from two SCO2‐mutated patients (babies) and two healthy donors (adults): (*i*) an Israeli male baby treated at the Rambam Health Care Campus in Haifa; (*ii*) an Estonian male baby treated at Tartu University Hospital in Estonia; (*iii*) a healthy adult Israeli female; (*iv*) a healthy adult Israeli male. Additionally, hair follicles were collected from a healthy adult Israeli female. iPSC were generated from the donors dermal fibroblasts (clones 5.2, 17.2, 24.5 and 27.10) or hair keratinocytes (clone N3) previously described 5. Following reprogramming, we used iPSC clones 17.2 and 27.10 generated from the Israeli and Estonian babies, accordingly. As control, we used clone 24.5 generated from a healthy 42‐year‐old female 5, 6. Additional control clones (5.2 and N3) were generated from healthy 25‐ and 23‐year‐old male and female, respectively. Clone FSE‐5 m was generated from an infant male and was used for β‐adrenergic responsiveness experiments. Unless stated otherwise, the control clone referred to in the article and Supporting information is 24.5 (HDF). Functional iPSC‐CMs were generated as previously described 7, 8, 9. Karyotyping, genotyping and pluripotency assay using teratoma formation assay are detailed in the Supporting information.

### Immunofluorescence staining

See details in the Supporting information.

### Measurements of intracellular [Ca^2+^]_i_ transients, contractions, extracellular electrograms, transmembrane action potentials and BRV

All methods and protocols are detailed in the Supporting information. In brief, [Ca^2+^]_i_ transients and contractions were measured from iPSC‐CMs composing embryoid bodies (EBs), using the IonOptix Calcium and Contractility system (Westwood, MA, USA), as previously described 5, 10. Transmembrane action potentials and extracellular electrograms were recorded by means of the whole cell patch clamp and microelectrode array (MEA), respectively 11, 12.

### Transmission electron microscopy of the skeletal muscle biopsy and iPSC‐CMs

The Israeli patient underwent a muscle (gastrocnemius) biopsy as part of the studies performed in patients with suspected mitochondrial disorder (see Supporting information for details). Transmission electron microscopy (TEM) analysis was performed on 15‐, 30‐ and 45‐day‐old (post‐plating) EBs from SCO2^G193S^ clone 17.2, SCO2^E140K^ clone 27.10 and control clone 24.5 as previously reported 13. See details in the Supporting information.

### Statistical analysis

Results are presented as mean ± S.E.M. See details in the Supporting information.

## Results

### The SCO2‐mutated patients

The Israeli patient was homozygote for a less common c.577G>A mutation in the SCO2 gene, termed SCO2^G193S^. This patient, hospitalized at the age of 3 months, had congenital stridor, encephalopathy, skeletal myopathy and lactic acidosis. Echocardiography and electrocardiography (ECG) performed at 4 months demonstrated major left ventricle hypertrophy (Fig. S1A and B). Electron microscopy of a muscle biopsy demonstrated glycogen accumulation and abnormal mitochondria (Fig. S1C and D). Enzyme assay indicated COX deficiency (1.2 K/mg) compared to normal values (16.81 ± 6.51 K/mg). The patient died at the age of 4 months due to cardiorespiratory failure. The Estonian patient had a compound heterozygote mutation c.418G>A in exon 2, termed SCO2^E140K^ and a heterozygote c.17INS19 bp mutation in the coding region. As previously described, the patient was diagnosed with fatal infantile cardioencephalomyopathy and died at the age of 13 weeks due to respiratory insufficiency. Abnormalities in mitochondria size and structure and low COX activity levels were found in fibroblasts and muscle biopsies 14.

### iPSC generation, characterization and differentiation into cardiomyocytes

The SCO2 iPSC clones from both patients expressed the pluripotent markers SSEA4, Oct4, TRA1‐60, Nanog, TRA1‐81 and Sox2, had normal karyotype, and demonstrated pluripotency by *in vivo* differentiation into derivatives of all three germ layers (Figs [Link], [Link], [Link]). In addition, we confirmed the homozygote switch of *G* to *A* at nucleotide 577 in the SCO2^G193S^ gene, the heterozygote switch of *G* to *A* at nucleotide 418 in one allele and the insertion c.17INS19 bp in the second allele of the SCO2^E140K^ gene (Figs S3 and 4). The healthy control clones 24.5 (HDF), 5.2 (HDF) and N3 (KT) were fully characterized previously (Fig. S7).

### Transmission electron microscopy analysis

**Figure 1:**
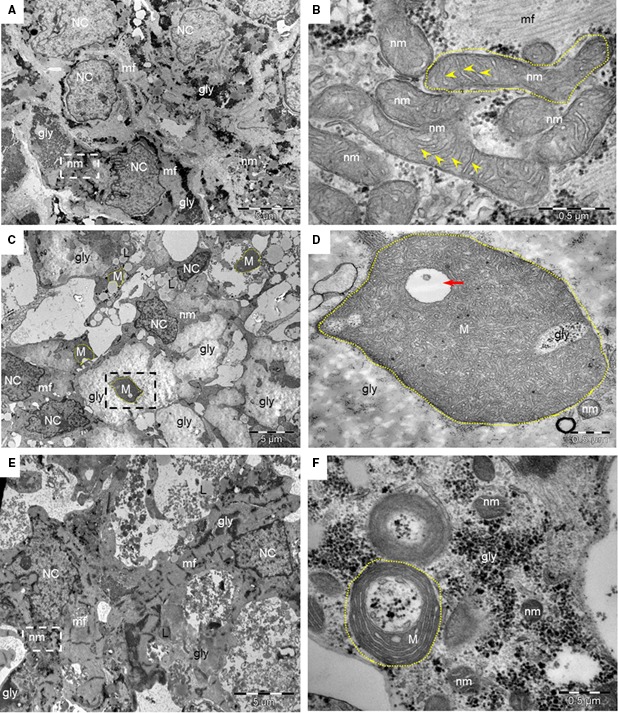
TEM images of control and SCO2‐mutated iPSC‐CMs. (**A**) Normal ultrastructure of 30‐day‐old control iPSC‐CMs with organized myofibrils (mf), grouped normal mitochondria (nm) and glycogen masses (gly). NC‐nucleus. (**B**) Higher magnification of the square marked area in (**A**) shows normal mitochondria (nm) from control iPSC‐CMs, containing parallel cristae (arrowheads). (**C**) 30‐day‐old SCO2^G193S^ iPSC‐CMs contain higher glycogen (gly) content, lipid droplets (L), poor organized myofibrils (mf) and oversized mitochondria (M). The intercellular space is enlarged compared with control. NC‐nucleus. (**D**) Enlarged mitochondrion (M) from 30‐day‐old SCO2^G193S^ iPSC‐CMs (square marked area in **C**) shows increased number of disarrayed cristae, vacuolated cristae (red arrow) and glycogen inclusions (gly). A normal mitochondrion (nm) is visible nearby. (**E**) 30‐day‐old SCO2^E140K^ cardiomyocytes show organized myofibrils (mf), large masses of glycogen (gly), lipid droplets (L) and clusters of mitochondria (nm). NC‐nucleus. (**F**) Higher magnification of the square marked area in **E** shows normal structured mitochondria (nm) and slightly enlarged, doughnut‐shaped mitochondria (M). gly: glycogen.

**Figure 2:**
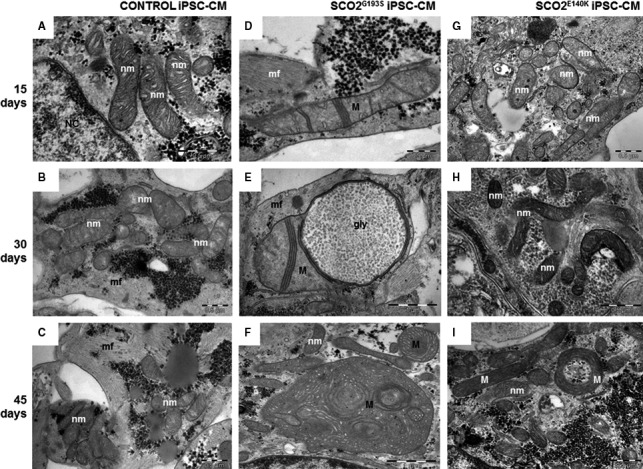
TEM images showing mitochondria from 15‐, 30‐ and 45‐day‐old control (**A–C**), SCO2^G193S^ (**D–F**) and SCO2^E140K^ (**G–I**) iPSC‐CMs. Normal mitochondria show few parallel cristae (nm). The mitochondria abnormalities (**M**) progress with the age of SCO2^G193S^ iPSC‐CMs: disarray and decreased number of mitochondrial cristae (**D**); few tightly packed cristae and glycogen accumulation inside the mitochondrion (**E**); mitochondria are enlarged and contain disarrayed and highly increased number of cristae (**F**). SCO2^E140K^iPSC‐CMs present mostly mitochondria with normal ultrastructure (nm) (**G‐I**) and rare doughnut‐like mitochondria (**M**) at 45 days (**I**), mf: myofibrils; NC: nuclei.

First, TEM analysis in 30‐day‐old control (Fig. 1A and B) and SCO2^G193S^ iPSC‐CMs (Fig. 1C and D) showed enlarged mitochondria, disarrayed mitochondrial cristae, intra‐mitochondrial vacuoles, large masses of glycogen, lipid droplets and doubled nuclei in the mutated cardiomyocytes. In contrast, SCO2^E140K^ iPSC‐CMs displayed only high quantities of glycogen and lipid droplets with minor mitochondrial ultrastructural changes (Fig. 1E and F; Fig. S5). While normal mitochondria had diameter of 0.30 ± 0.06 μm (*n* = 50), in SCO2^G193S^ iPSC‐CMs mitochondria were much larger—0.50 ± 0.37 μm (*n* = 50, *P* < 0.001 *versus* control) (Table S1). Next, we found that ageing (15, 30 and 45 days) of SCO2^G193S^ iPSC‐CMs was associated with progression of the ultrastructural mitochondrial abnormalities compared with mitochondria from control and SCO2^E104K^ iPSC‐CMs. While 15‐day‐old cardiomyocytes showed mild mitochondrial abnormalities (Fig. 2D), 30‐day‐old cardiomyocytes demonstrated abnormally large mitochondria with few tightly packed cristae and intra‐mitochondrial glycogen accumulation (Fig. 2E). Accordingly, 45‐day‐old cardiomyocytes displayed oversized mitochondria, with disarrayed and highly increased number of curled cristae (Fig. 2F).

### Functional characteristics and responsiveness to positive inotropic interventions

The first step in this series of experiments was to determine whether the mutated cardiomyocytes have different [Ca^2+^]_i_ transient and contraction characteristics than control cardiomyocytes. As seen in Figure S6, all the functional characteristics were similar in the three experimental groups, suggesting that the mutation did not affect the basal excitation–contraction coupling machinery. Next, to test our working hypothesis, we investigated the inotropic effects of β‐adrenergic stimulation (with isoproterenol), increased [Ca^2+^]_o_ and AT‐II.

#### β‐adrenergic stimulation

**Figure 3:**
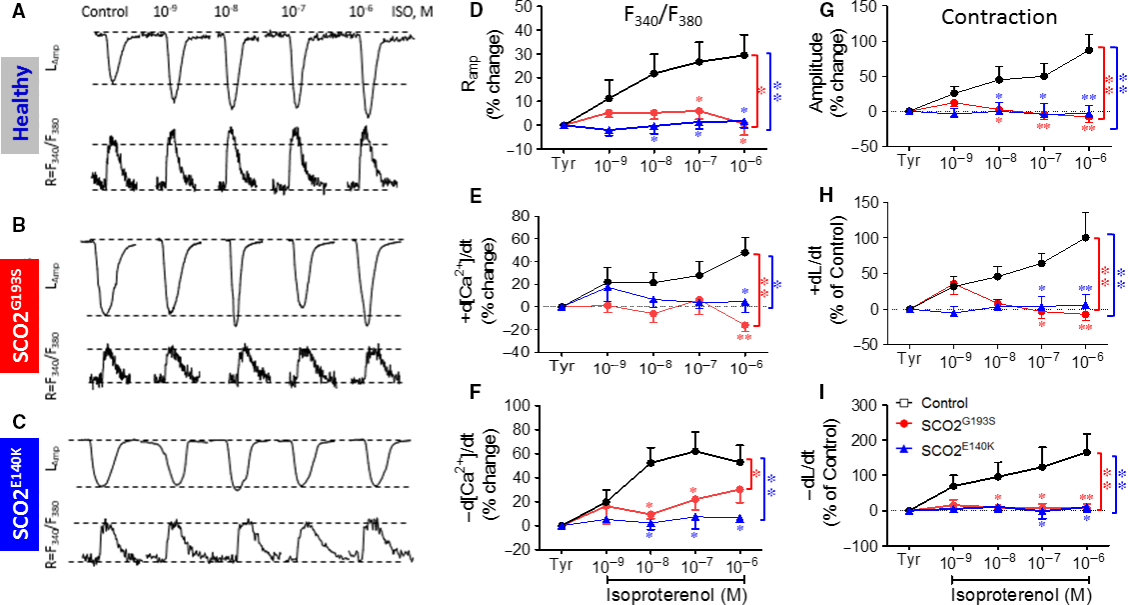
The effects of isoproterenol on [Ca^2+^]_i_ transients and contractions in control, SCO2^G193S^ and SCO2^E140K^ iPSC‐CMs. (**A–C**) [Ca^2+^]_i_ transients (R = F_340_/F_380_) and contractions (*L*_amp_) from control, SCO2^G193S^ and SCO2^E140K^ iPSC‐CMs, respectively. (**D**) [Ca^2+^]_i_ transient amplitude (*R*_amp_); (**E**) maximal rate of [Ca^2+^]_i_ rise (+d[Ca^2+^]/dt); (**F**) maximal rate of [Ca^2+^]_i_ decay (‐d[Ca^2+^]/dt); (**G**) maximal amplitude (*L*_amp_); (**H**) maximal contraction rate (+dL/dt); (**I**) maximal relaxation rate (−dL/dt). The effect of isoproterenol on contraction characteristics of SCO2^G193S^ (*n* = 13), SCO2^E140K^ (*n* = 6) and control (*n* = 8) was expressed as per cent change. The effect of isoproterenol on the [Ca^2+^]_i_ transients was expressed as the per cent change in the fluorescence ratio, F_340_/F_380_ (*n* = 6, *n* = 5 and *n* = 10, for SCO2^G193S^, SCO2^E140K^ and control respectively); Tyr: Tyrode's solution; *P < 0.05, **P < 0.001 (*versus* control).

A fundamental cardiac feature is β‐adrenergic‐mediated positive inotropy caused by increased SR Ca^2+^ release, which relies on the ATP‐dependent SERCA activity 15. As shown in Figure 3, isoproterenol caused marked dose‐dependent positive inotropic and lusitropic effects in control iPSC‐CMs (*n* = 18, *P* < 0.05). Importantly, similar effects were observed in other control clones including 5.2 (HDF) and N3 (KT) (Fig. S8). In contrast, SCO2^G193S^ (*n* = 19) and SCO2^E140K^ (*n* = 11) iPSC‐CMs were completely unresponsive to isoproterenol (*P* < 0.05 compared to control).

#### Effects of increased [Ca^2+^]_o_

**Figure 4:**
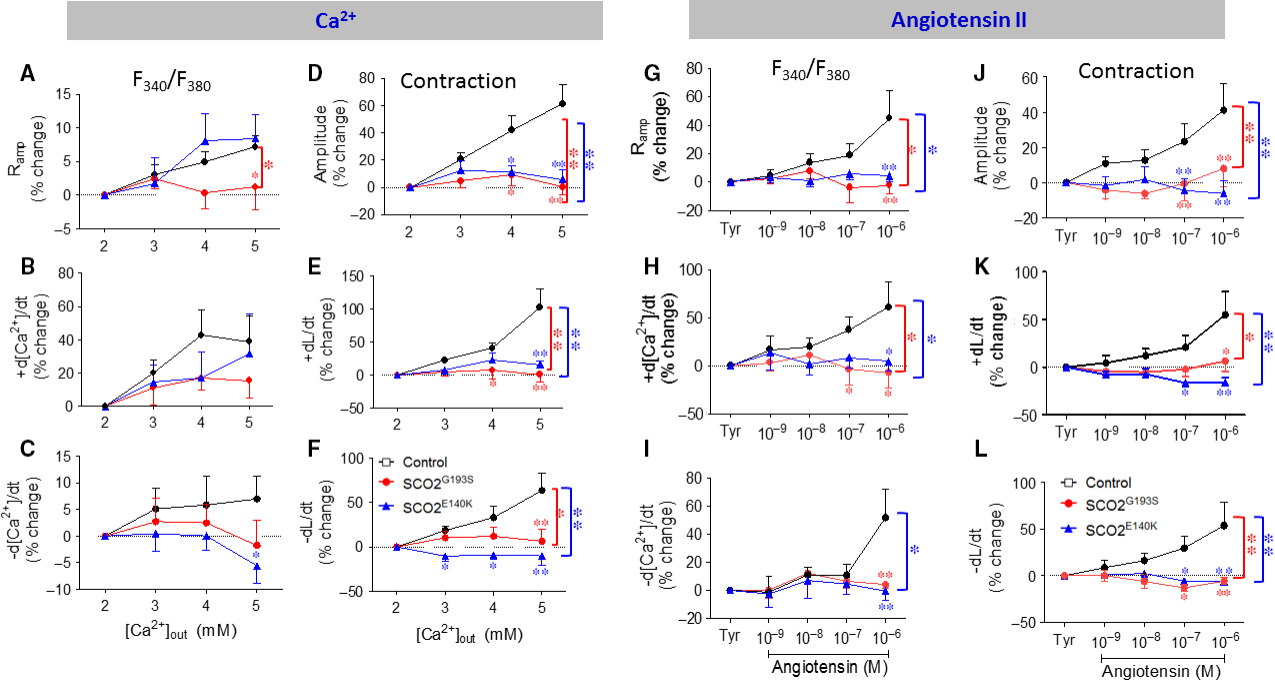
The effects of increased [Ca^2+^]_o_ on the [Ca^2+^]_i_ transients and contractions. (**A**) R_amp_; (**B**) +d[Ca^2+^]/dt; (**C**) −d[Ca^2+^]/dt; (**D**) L_amp_; (**E**) +dL/dt; (**F**) −dL/dt. The effect of [Ca^2+^]_o_ on contraction characteristics of SCO2^G193S^ (n = 6), SCO2^E140K^ (n = 7) and control (n = 12) was expressed as per cent change. The effects of AT‐II on the [Ca^2+^]_i_ transients and contractions. (**G**) R_amp_; (**H**) +d[Ca^2+^]/dt; (**I**) ‐d[Ca^2+^]/dt; (**J**) L_amp_; (**K**) +dL/dt; (**L**) −dL/dt. The effect of AT‐II on contraction characteristics of SCO2^G193S^ (n = 7), SCO2^E140K^ (n = 8) and control (n = 10) was expressed as percent change. The effect on the [Ca^2+^]_i_ transients was expressed as the percent change in the fluorescence ratio, F_340_/F_380_ (n = 6, n = 6 and n = 7, for SCO2^G193S^, SCO2^E140K^ and control respectively); Tyr: Tyrode's solution; *P < 0.05, **P < 0.001 (versus control).

To decipher whether the attenuated response to isoproterenol was due to dysfunctional β‐adrenergic cascade or alternatively—impaired downstream element mediating any positive inotropic intervention, we investigated the effect of elevating [Ca^2+^]_o_; this intervention augments L‐type Ca^2+^ current (I_Ca,L_), which in turn increases SR Ca^2+^ release thereby increasing contractile force 16. Indeed, as shown in Figure 4A–C, in control cardiomyocytes (*n* = 12), elevating [Ca^2+^]_o_ caused positive inotropic and lusitropic effects (*P* < 0.001), although the [Ca^2+^]_i_ transients were affected to a lesser extent than contractions (Fig. 4D–F). In contrast, in agreement with their depressed response to isoproterenol, both SCO2^G193S^ and SCO2^E140K^ iPSC‐CMs were completely unresponsive (*P* < 0.05 *versus* control) to increased [Ca^2+^]_o_.

#### Effects of AT‐II

Based on the above findings, our interim conclusion was that the common downstream denominator of these positive inotropic interventions is a defective SR in the mutated iPSC‐CMs. To support this notion, we investigated whether AT‐II, also inducing its positive inotropy *via* SR‐Ca^2+^ 17, is equally ineffective in the mutated cardiomyocytes. Indeed, whereas in control iPSC‐CMs AT‐II caused prominent dose‐dependent positive inotropic and lusitropic effects (*n* = 17, *P* < 0.05), SCO2^G193S^ (*n* = 13) and SCO2^E140K^ (*n* = 14) iPSC‐CMs were unresponsive (Fig. 4G–L). Hence, except for the maximal rate of [Ca^2+^]_i_ decay which was not different between control and SCO2^G193S^ iPSC‐CMs (significant only at 10^−6^M, *P* < 0.001), all control dose‐response relations were significantly (*P* < 0.05) different from the mutated cardiomyocytes.

#### Responsiveness to caffeine

**Figure 5:**
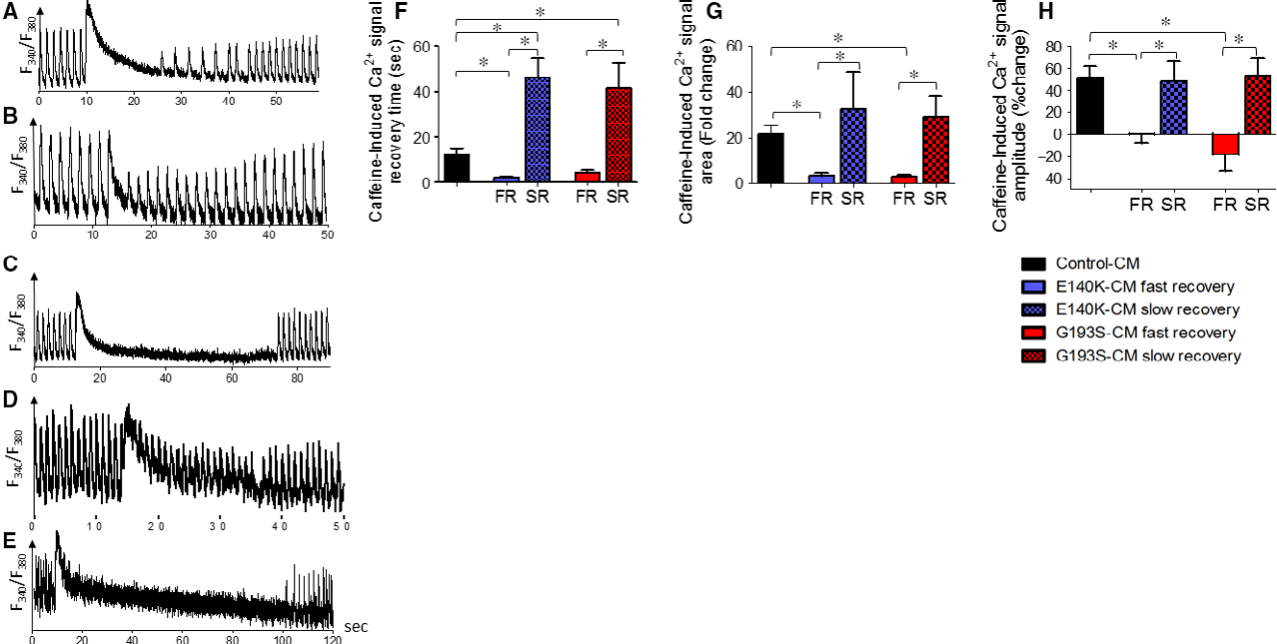
The effects of caffeine on control, SCO2 ^E140K^ and SCO2^G193S^ iPSC‐CMs. [Ca^2+^]_i_ transients from (**A**) control (**B–C**) SCO2^E140K^ and (**D–E**) SCO2^G193S^ iPSC‐CMs, demonstrating the effect of caffeine. (**F**) The mean recovery time, calculated as the time from the peak of caffeine‐induced [Ca^2+^]_i_ rise to the first measurable [Ca^2+^]_i_ transient; (**G**) per cent change in area of the caffeine‐induced Ca^2+^ signal compared to the pre‐caffeine area; (**H**) per cent change in caffeine‐induced Ca^2+^ signal amplitude compared to the pre‐caffeine amplitude. Control iPSC‐CMs (*n* = 7), SCO2^G193S^ iPSC‐CMs (*n* = 11), SCO2^E140K^ iPSC‐CMs (*n* = 20), *P < 0.05. Asterisk above bars connecting columns represents significant difference between groups. Each SCO2‐mutated group was divided into subgroups FR and SR according to type of reaction.

Because the SR is the common denominator of all three positive inotropic interventions, we tested its functionality using a brief application of caffeine (10 mM) which opens RyR2 channels and releases SR Ca^2+^. In agreement with our previous report 5, in control cardiomyocytes, caffeine caused an abrupt increase in [Ca^2+^]_i_ concomitant with contraction cessation, followed by a gradual decline in [Ca^2+^]_i_ along with resumption (within ~13 sec.) of contractions (Fig. 5A). In contrast, in SCO2^E140K^ cardiomyocytes (Fig. 5B and C), the response to caffeine was different, and included two major types, with equal proportions. In 45% of the experiments (total of 22), the response (termed ‘fast recovery’, FR) was smaller and shorter than control, and contractions resumed within ~0.8–5 sec. after caffeine application (Fig. 5B and F). In 45% of cardiomyocytes, the response amplitude was similar to control but recovery was much slower, within 21–89 sec. (Fig. 5C and F); this group was termed ‘slow recovery’ (SR). The remaining 10% of the SCO2^E140K^ mutated iPSC‐CMs displayed a healthy‐like behaviour. Similarly (Fig. 5D and E), 31% of the SCO2^G193S^ cardiomyocytes (total of 13) had FR, 54% SR and 15% control‐like response. To quantify the response to caffeine, we calculated three parameters: (*i*) recovery time—the time from the peak of caffeine‐induced [Ca^2+^]_i_ rise to the first transient; (*ii*) the per cent change in caffeine‐induced [Ca^2+^]_i_ signal amplitude, compared to pre‐caffeine amplitude; and (*iii*) the per cent change in caffeine‐induced Ca^2+^ signal area, compared to the pre‐caffeine value. In the SR group of both SCO2‐mutated cardiomyocytes, the recovery time was longer (*P* < 0.05), and the area and amplitude were similar to control cells (Fig. 5F–H). In the FR group, the SCO2^G193^ cells showed similar recovery time to control, whereas SCO2^E140K^ displayed shorter recovery time (*P* < 0.05; Fig. 5F). In both mutations, the area and amplitude were smaller than control (*P* < 0.05; Fig. 5G–H). In summary, the depressed SR Ca^2+^ release capacity of the SCO2‐mutated iPSC‐CMs is likely to underlie the attenuated positive inotropic responsiveness to increased isoproterenol, [Ca^2+^]_o_ and AT‐II.

### Arrhythmias in the SCO2‐mutated cardiomyocytes

#### Transmembrane action potentials

**Figure 6:**
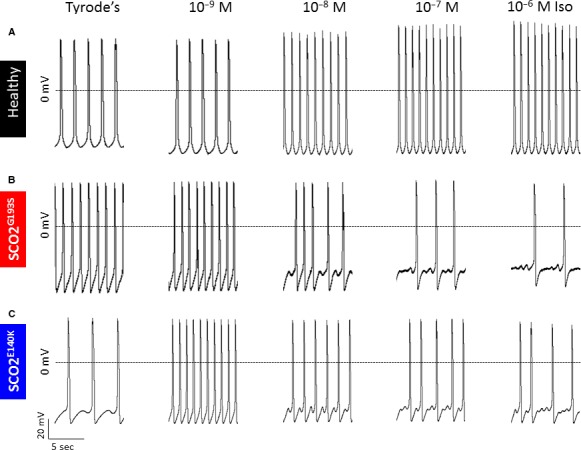
Transmembrane action potential recordings from control, SCO2^G193S^ and SCO2^E140K^ iPSC‐CMs in response to increasing concentrations of isoproterenol. (**A**) Representative recordings from control iPSC‐CMs in the presence of Tyrode's solution and isoproterenol concentrations of 10^−9^ M, 10^−8^ M, 10^−7^ M and 10^−6^ M. Control iPSC‐CMs displayed a positive chronotropic response to increasing concentrations of isoproterenol, represented by firing rate. (**B**) Recordings from SCO2^G193S^ and (**C**) from SCO2^E140K^ iPSC‐CMs in the presence of Tyrode's solution and increasing isoproterenol concentrations. SCO2^G193S^ iPSC‐CMs show generation of DADs with isoproterenol. SCO2^E140K^ iPSC‐CMs display DADs under baseline conditions and in the presence of different isoproterenol concentrations.

The results obtained thus far suggest that in the mutated cardiomyocytes, the SR is at least partially depleted of Ca^2+^, implying that the cytoplasm is Ca^2+^ overloaded, which can thus give rise to delayed afterdepolarizations (DADs) and triggered arrhythmias 10. To determine whether the mutated cardiomyocytes exhibit arrhythmias typical of Ca^2+^ overload, transmembrane action potentials were recorded from small clusters of cardiomyocytes in the absence and presence of increasing isoproterenol concentrations. As depicted in Figure 6A, in control cardiomyocytes, isoproterenol caused a typical dose‐dependent positive chronotropic effect. In contrast (in agreement with the Ca^2+^ overload concept), in SCO2^E140K^ and SCO2^G193S^ cardiomyocytes (Fig. 6B and C), DADs began to appear under baseline conditions (two of three experiments) or in the presence of low (10^−8^ M) (2/3) isoproterenol concentration, respectively. In addition to DADs, in mutated cardiomyocytes, increasing isoproterenol concentrations led to decreased firing rate (from 10^−8^ M) and oscillatory pre‐potentials (starting from 10^−8 ^M and 10^−7 ^M in SCO2^G193S^ and SCO2^E140K^ cardiomyocytes, respectively) (4/6). Interestingly, in one of the experiments with SCO2^E140K^ cardiomyocytes, 10^−9^ M isoproterenol markedly increased the firing rate and eliminated the DADs, but then starting from 10^−8^ M prominent DADs appeared along with a decline in the firing rate (Fig. 6C).

#### Extracellular electrograms recorded from contracting cardiomyocyte networks

**Figure 7:**
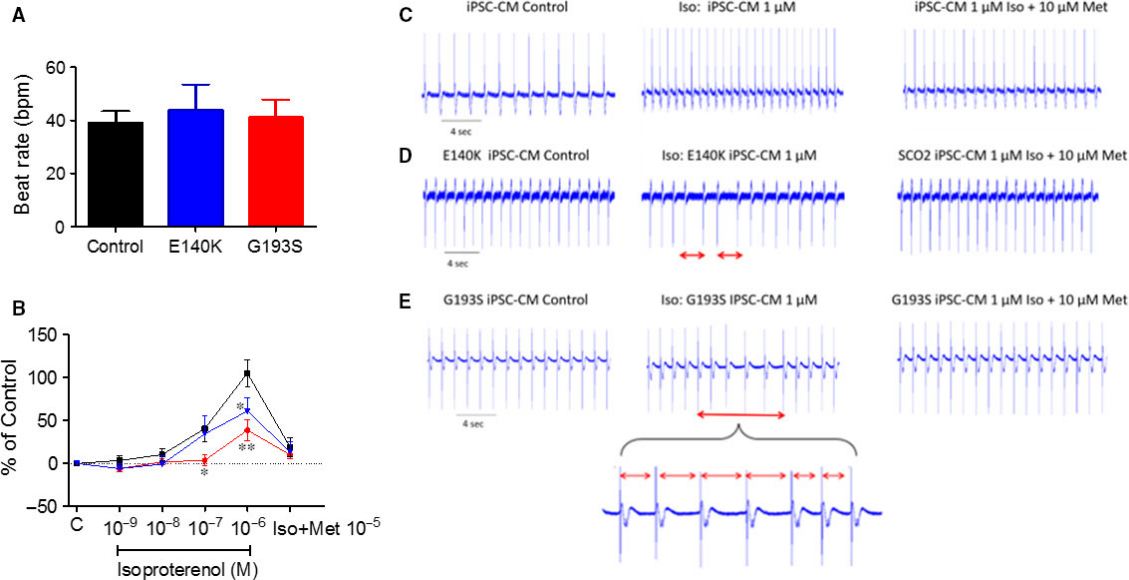
The chronotropic response of extracellular electrograms recorded from control, SCO2^E140K^ and SCO2^G193S^ iPSC‐CMs to isoproterenol. (**A**) The spontaneous beating rate of control (*n* = 11), SCO2^E140K^ (*n* = 7) and SCO2^G193S^ (*n* = 10) iPSC‐CMs. (**B**) The effect of isoproterenol on the beat rate of iPSC‐CMs and the blocking effect of the β‐blocker metoprolol. (**C–E**) The EB was perfused initially with DMEM solution (Control) and then with isoproterenol 10^−6^ M in DMEM solution. The different time intervals denote the arrhythmia in the isoproterenol‐treated SCO2‐mutated EBs. The arrhythmia was blocked by the β‐blocker metoprolol. (**C**) Control, (**D**) SCO2^E140K^ and (**E**) SCO2^G193S^ iPSC‐CMs. Results are expressed as per cent change from control (absence of isoproterenol). The arrhythmia is marked by red bar; bpm: beats per minute; C: control conditions—DMEM solution in the absence of isoproterenol; Iso: Isoproterenol; Met: Metoprolol; *P < 0.05, **P < 0.001 (*versus* control).

To support the arrhythmogenic findings at the single cell level, we confirmed the presence of isoproterenol‐induced arrhythmias in a network of contracting cardiomyocytes, by recording extracellular electrograms using the MEA system, in the absence and presence of isoproterenol. In the absence of isoproterenol, the spontaneous beat rate was similar in the three experimental groups (Fig. 7A), suggesting the basic pacemaker machinery was not affected by the SCO2 mutations. In agreement with the reduced inotropic and chronotropic responsiveness to isoproterenol (Figs. 3 and 6), the mutated cardiomyocytes from both patients exhibited an attenuated positive chronotropic response (Fig. 7B) compared to control cardiomyocytes. In these experiments, the cells were initially perfused for 30 min. with serum‐free DMEM solution at 37°C, followed by 10 min. of perfusion with increasing concentrations of isoproterenol (10^−9^ M–10^−6^M). Concomitant with the smaller chronotropic response, isoproterenol (10^−6^M) caused arrhythmias expressed by the irregular spontaneous firing rate (Fig. 7C–E), compatible with the arrhythmia at the single cell level. As expected, these arrhythmias were blocked by metoprolol. These findings were repeated in 9 control and 15 mutated cardiomyocytes.

#### BRV characteristic in SCO2‐mutated *versus* control cardiomyocytes

**Figure 8:**
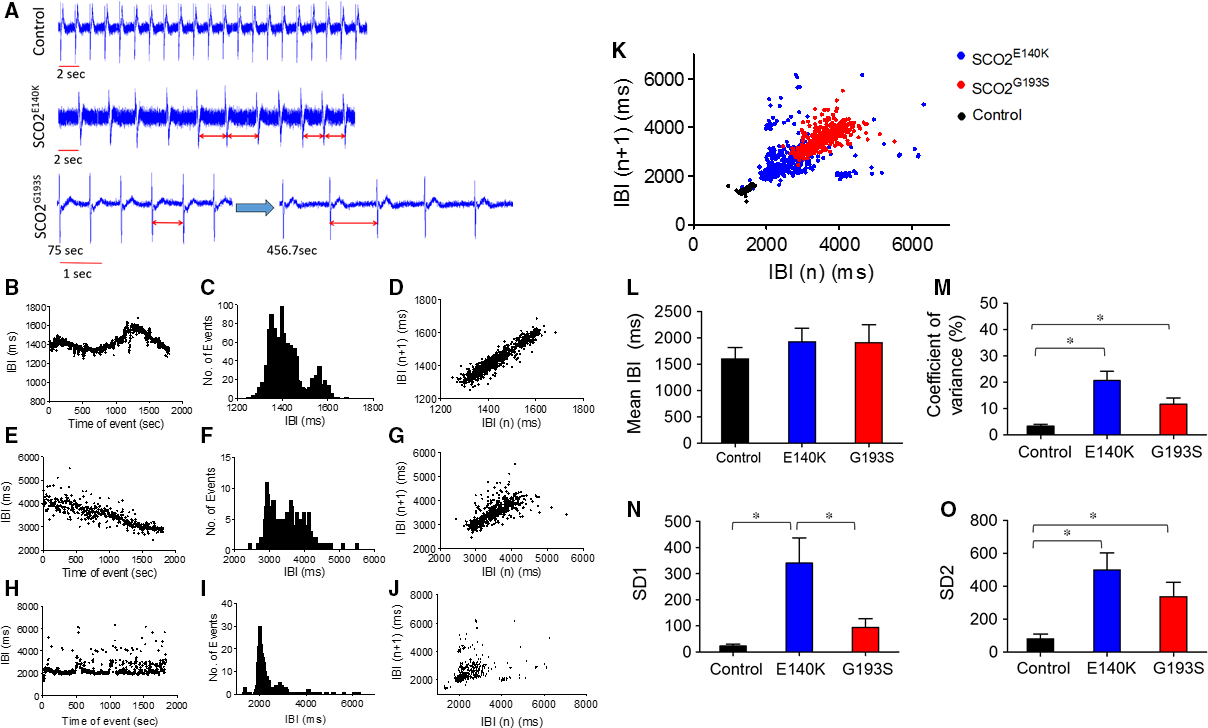
The spontaneous electrical activity and BRV properties of control, SCO2^E140K^ and SCO2^G193S^ iPSC‐CMs. (**A**) EBs were perfused with DMEM solution, activation spikes and repolarization waves were recorded in control, SCO2^E140K^ and SCO2^G193S^ iPSC‐CMs (first recorded at 75 sec. and then at 456 sec.). Inter‐beat intervals (IBIs), histograms and Poincaré plots analysis in control (**B–D**), SCO2^G193S^ (**E–G**) and SCO2^E140K^ iPSC‐CMs (**H–J**). (**B, E, H**) IBIs time series, (**C, F, I**) Histogram distribution of IBIs, (**D, G, J**) Poincaré plots of the BRV. (**K**) Combined Poincaré plots of (**D, G, J**). (**L–O**) Comparisons of BRV magnitude in control (*n* = 8), SCO2^E140K^ (*n* = 5) and SCO2^G193S^ (*n* = 8). (**L**) Summary of mean IBI, (**M**) coefficient of variance of IBIs (IBI CV), (**N**) SD1 and (**O**) SD2 of Poincaré plots in contracting EBs. Altered time intervals were marked by red bar. *P < 0.05.

As we previously demonstrated that disturbed intracellular Ca^2+^ handling (likely to occur in the SCO2‐mutated cardiomyocytes) augments the BRV indices 11, 12, we compared the BRV characteristic in mutated *versus* control cardiomyocytes. To this end, spontaneous electrograms were recorded for 30 min., and BRV analysis performed in the three experimental groups. The first indication of increased BRV in the mutated cardiomyocytes is demonstrated by the dissimilar inter‐beat intervals (IBIs), compared to the fixed intervals in the control cells (Fig. 8A). The increased BRV in the mutated cardiomyocytes is further illustrated by the IBI *versus* time plots (Fig. 8B, E and H), and the histograms (Fig. 8C, F and I) depicting the number of events at different interval ranges. As shown by the histograms, the range of IBIs was broader in SCO2 mutated than in control cardiomyocytes. Specifically, while in control cells, the IBIs range was ~300 msec, the ranges in the SCO2^G193S^ and the SCO2^E140K^ were ~2000 msec. and in 2000–3000 msec., respectively. Accordingly, the IBIs coefficient of variation (CV) values were larger (*P* < 0.05) in the mutated than in control cardiomyocytes (Fig. 8M).

The BRV magnitude was further analysed by generating Poincaré plots and calculating SD1 and SD2 representing the standard deviation (S.D.) of short‐term and long‐term IBI variability, respectively. In support of the higher CV values, the representative (Fig. 8D, G and J) and superimposed Poincaré plots (Fig. 8K) illustrate the much larger dispersion of the data points in the mutated cardiomyocytes. Accordingly, except for the mean IBI that showed no significant difference between control and mutated clones (Fig. 8L) and for SD1 which was not different between control and SCO2^G193S^ iPSC‐CMs, the Poincaré plot indices were higher in the mutated (from both patients) than in the control cardiomyoctes (Fig. 8M–O).

## Discussion

Mutations in the SCO2 gene are among the most common causes of COX deficiency 18 leading to HCM, heart and respiratory failure, and death at infancy 19. To investigate the mechanisms underlying the pathological features of the affected cardiomyocytes, we tested the hypothesis that the SCO2 mutation is associated with mitochondrial abnormalities, and intracellular Ca^2+^‐overload resulting in functional derangements and arrhythmias. The major findings in SCO2 cardiomyocytes were as follows: (*i*) ultrastructural abnormalities, mainly enlarged mitochondria and distorted cristae; (*ii*) attenuated inotropic response to β‐adrenergic stimulation, [Ca^2+^]_o_ and AT‐II; (*iii*) abnormal responses to caffeine administration; and (*iv*) DADs and triggered arrhythmias.

### Ultrastructural abnormalities in SCO2‐mutated cardiomyocytes

Comprehensive TEM analysis in SCO2^G193S^ cardiomyocytes demonstrated age‐dependent (15‐, 30‐ and 45‐day‐old cultures) abnormalities including enlargement of mitochondria, disorganized mitochondrial cristae, intra‐mitochondrial vacuoles, glycogen deposits and lipid droplets. In contrast, SCO2^E140K^ cardiomyocytes had normal mitochondrial distribution and structure, but large cellular glycogen deposits and lipid droplets. Currently, there is no explanation for these differences; other than that mitochondrial abnormalities seen in mitochondrial diseases are generally heterogeneous and there is often no clear genotype–phenotype correlation. Hence, the mutations in SCO2 are in different domains of the protein; the E140K mutation is close to the copper binding site, while the G193S mutation is in a different segment of the protein, which may also result in different effects of the mutations.

Mitochondrial structural abnormalities, including massive proliferation, are a secondary effect of the complex IV deficiency, and there is no universal explanation for how these structural changes come about. Possibly, ATP shortage due to COX deficiency in the oxidative phosphorylation pathway leads to a compensatory response causing swelling of mitochondria in an effort to produce more ATP. Further, mitochondrial proliferation is often (but certainly not always) seen in mitochondrial diseases, and it is thought to be a compensatory mechanism involving PGC1 alpha/AMPK signalling 20. Glycogen deposits are typical in SCO2‐mutated human cells 21. Notably, the TEM findings in cardiomyocytes are in agreement with the pathological findings demonstrated in the muscle biopsies taken from the hospitalized patients who showed mitochondrial abnormalities and low COX activity. This similarity further strengthens the validity of our model as an *in vitro* recapitulation of the *in vivo* disease.

### Attenuated response of SCO2‐mutated iPSC‐CMs to positive inotropic interventions

In agreement with our previous studies and others 5, 17, 22 in control iPSC‐CMs, β‐adrenergic stimulation, increased [Ca^2+^]_o_ and AT‐II caused prominent positive inotropic and lusitropic effects (Fig. 3 and 4). In marked contrast to the control responses, the mutated cardiomyocytes from both patients were completely unresponsive to all three positive inotropic interventions. As will be discussed below, the most probable explanation for these findings is that a downstream common step in these different inotropic cascades is impaired in SCO2‐mutated cardiomyocytes. In brief, higher [Ca^2+^]_o_ augments the Ca^2+^ chemical gradient, leading to increased I_Ca,L_ amplitude, resulting in enhanced SR Ca^2+^ release which increases the contractile force. Isoproterenol activates the β‐adrenergic cascade which *via* increased protein kinase A (PKA) activation, augments I_Ca,L_ which enhances SR Ca^2+^ release, thereby augmenting contraction 15. On the other hand, AT‐II induces a positive inotropic effect by binding to its respective membrane receptor, and activating phospholipase C (PLC) 17, which synthesizes 1,4,5‐IP_3_, in turn increasing intracellular Ca^2+^ by opening SR 1,4,5‐IP_3_‐dependent Ca^2+^ channels. Collectively, the common denominator of these three positive inotropic interventions is increased SR Ca^2+^ release.

### The mechanism underlying the attenuated positive inotropic responsiveness

To test the hypothesis that impaired SR Ca^2+^ content/release is responsible for the attenuated inotropic responses, we tested the effect (in control and SCO2‐mutated cardiomyocytes) of caffeine which releases Ca^2+^ from the SR by reducing the activation threshold of RyR2 23. Indeed, as illustrated in control cardiomyocytes, in agreement with our previous study 5 caffeine induced a long‐lasting rise in [Ca^2+^]_i_ along with a transient depression of the contractions, followed by a gradual resumption of contractions. In contrast, the responses of mutated cardiomyocytes were completely different, featuring two major phenomena. Both phenotypes depicted a small and short (compared to control) Ca^2+^ spike: the first phenotype showed a FR and the second phenotype depicted a SR of contractions. The residual 10–20% (depending on the mutation) exhibited a control‐like phenotype. Thus, while control cardiomyocytes depicted a single typical response to caffeine, the mutated cells exhibited dissimilar (two types) responses to caffeine. These two abnormal responses of the mutated cardiomyocytes were demonstrated previously in sarcomeric‐HCM mutated cardiomyocytes 24.

**Figure 9:**
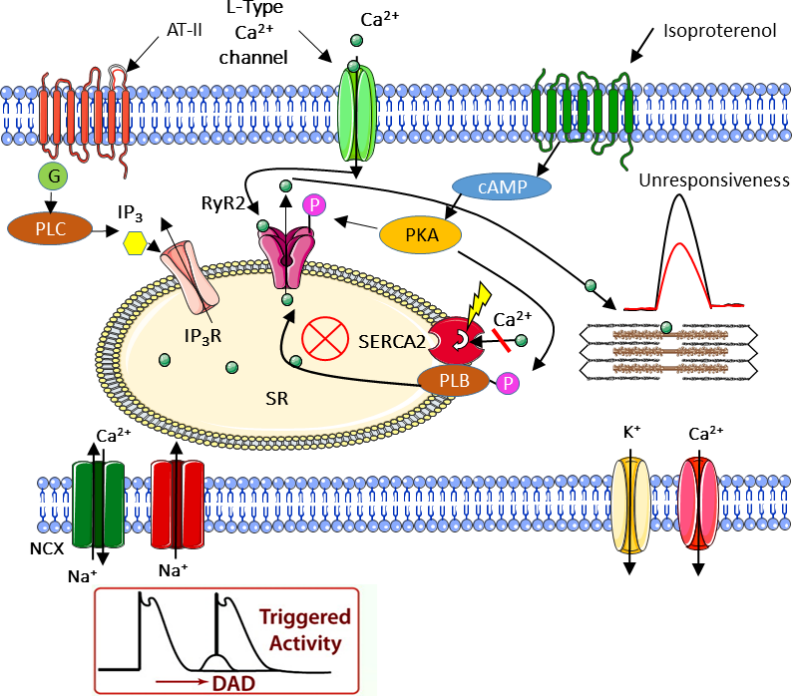
Schematic model of a proposed mechanism for the observed abnormalities in SCO2 iPSC‐CMs. The scheme describes the three distinct cellular pathways converging at the sarcoplasmic reticulum (SR). The scheme suggests an explanation for the delayed afterdepolarizations (DADs) and the attenuated inotropic responsiveness of SCO2‐mutated cardiomyocytes. While in control cardiomyocytes, these different pathways cause positive inotropic and lusitropic effects due to SR Ca^2+^ release, in SCO2‐mutated iPSC‐CMs, Ca^2+^ depleted SR accounts for the impaired inotropic responses.

While currently we cannot offer an explanation for the SR and FR phenotypes, it is likely they are both caused by disturbed intracellular Ca^2+^ handling resulting from the SCO2 mutation and COX impairment. Specifically, the most likely element underlying these Ca^2+^ handling abnormalities is the ATP‐dependent SERCA pump, responsible for Ca^2+^ re‐uptake into the SR 25, 26, and thus ATP shortage is expected to reduce its activity. Therefore, the depressed ATP synthesis and consequently reduced SERCA activity results in lower SR Ca^2+^ stores, leading to attenuated RyR2‐mediated Ca^2+^‐induced‐Ca^2+^‐release. Collectively, whereas the three positive inotropic stimuli tested (β‐adrenergic stimulation, increased [Ca^2+^]_o_ and AT‐II) augment contraction *via* different pathways (Fig. 9), they share one common downstream element, which is impaired in SCO2‐mutated cardiomyocytes—ATP‐dependent SR Ca^2+^ storage capacity. Finally, an important observation that needs to be reconciled with the attenuated positive inotropic response is the control‐like basal [Ca^2+^]_i_ transient and contraction characteristics in the SCO2‐mutated cardiomyocytes (Fig. S5). These results suggest that under unstressed conditions, the basal SR Ca^2+^ content maintained by the existing ATP levels (produced by a combination of glycolysis and oxidative phosphorylation) is sufficient to support the excitation‐contraction coupling machinery 26. However, these depressed SERCA‐dependent Ca^2+^ levels are insufficient to provide the excessive Ca^2+^ required to generate a positive inotropic effect. Finally, our findings are consistent with previous studies demonstrating ATP shortage as a cause of HCM 27, 28, 29, 30. Low ATP levels lead to abnormally high levels of cytosolic Ca^2+^ due to impaired SERCA activity. The high Ca^2+^ concentration activates various downstream proteins and pathways including Ca^2+^/calmodulin‐dependent protein phosphatase (calcineurin), different nuclear factors of activated T cells (NFAT), Ca^2+^/calmodulin‐dependent protein kinase (CaMK) and p38 mitogen‐activated protein kinase, which eventually results in HCM and cardiac dysfunction 31.

As depicted in Figure 9, we propose a unified theory underlying the described abnormalities in SCO2 iPSC‐CMs. The attenuated response of the mutated cardiomyocytes to positive inotropic stimulation (in the form of isoproterenol, AT‐II and Ca^2+^ administration) all indicate abnormalities in different cellular pathways converging at the SR. Together with damaged mitochondria demonstrated by TEM analysis, we singled out SERCA, the ATP‐dependent pump responsible for Ca^2+^ re‐uptake into the SR, as a suspected dysfunctional key element resulting in intracellular Ca^2+^ overload. In agreement with this conclusion, spontaneous action potential recordings demonstrated DADs, known to originate from Ca^2+^ overload as well.

In summary, collectively, our novel findings reveal abnormal intracellular Ca^2+^ handling in SCO2‐mutated cardiomyocytes, accounted for by ATP shortage due to the impaired mitochondrial respiratory chain function. These findings are consistent with other HCM iPSC‐CMs studies which demonstrated irregular Ca^2+^ handling as a fundamental mechanism underlying the disease pathology 32. However, in this study, we present for the first time Ca^2+^ abnormalities as a prominent aspect of mitochondrial‐related HCM. Furthermore, the similar properties of different HCM subtypes may indicate a basic common mechanism to which pathological Ca^2+^ handling can be attributed. Additional research is required to determine whether a common mechanism is indeed responsible for the similar phenotypes or perhaps the resembling outcomes originate from different causes leading to the same HCM disease.

## Conflict of interest

None declared.

## Supporting information


**Figure S1** Clinical data of the SCO2 patient at 4 months of age (prior to death).

**Figure S2** Pluripotency of iPSC derived from the SCO2 patient and a healthy control.

**Figure S3** Genetic, immunofluorescence and histological characterization of SCO2 iPSC.

**Figure S4** Genetic and histological characterization of iPSC derived from SCO2 patients and a healthy control.

**Figure S5** Transmission electron microscopy (TEM) demonstrating ultrastructural abnormalities in SCO2 iPSC‐CM.

**Figure S6** Basal [Ca^2+^]_i_ transients and contractions in control and SCO2 iPSC‐CMs.

**Figure S7** Pluripotency of iPSC derived from human dermal fibroblasts and hair keratinocytes.

**Figure S8** Effect of isoproterenol on [Ca^+2^]_i_ transients and contractions in KT and HDF iPSC‐CMs.

**Table S1**. Mean diameter of mitochondria in control and SCO2‐mutated iPSC‐CMs.
