# Supplementary figures and images for: Investigating the cardiac pathology of SCO2‐mediated hypertrophic cardiomyopathy using patients induced pluripotent stem cell–derived cardiomyocytes
Source: J Cell Mol Med. 2017 Nov 28;22(2):913–25. doi: 10.1111/jcmm.13392 (PMC5783844; doi:10.1111/jcmm.13392)

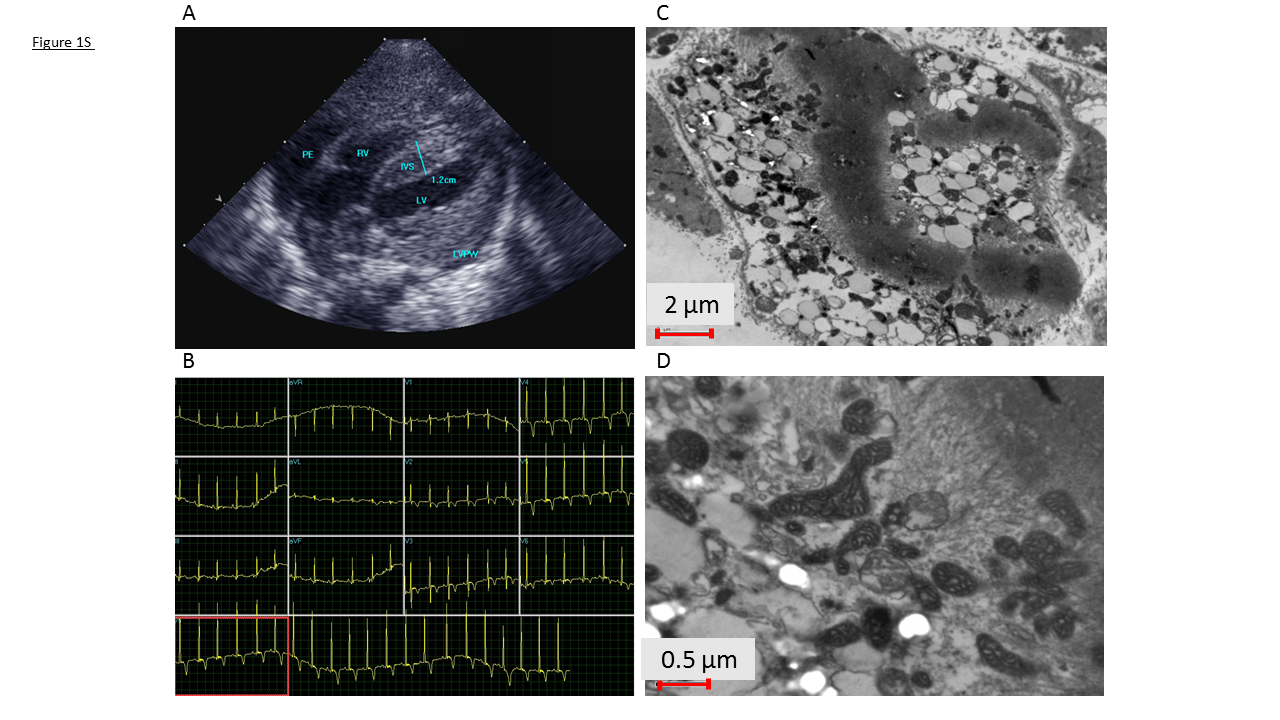

Supplement: Supplementary file 1 — Figure S1 Clinical data of the SCO2 patient at 4 months of age (prior to death). [file JCMM-22-913-s001.tif]

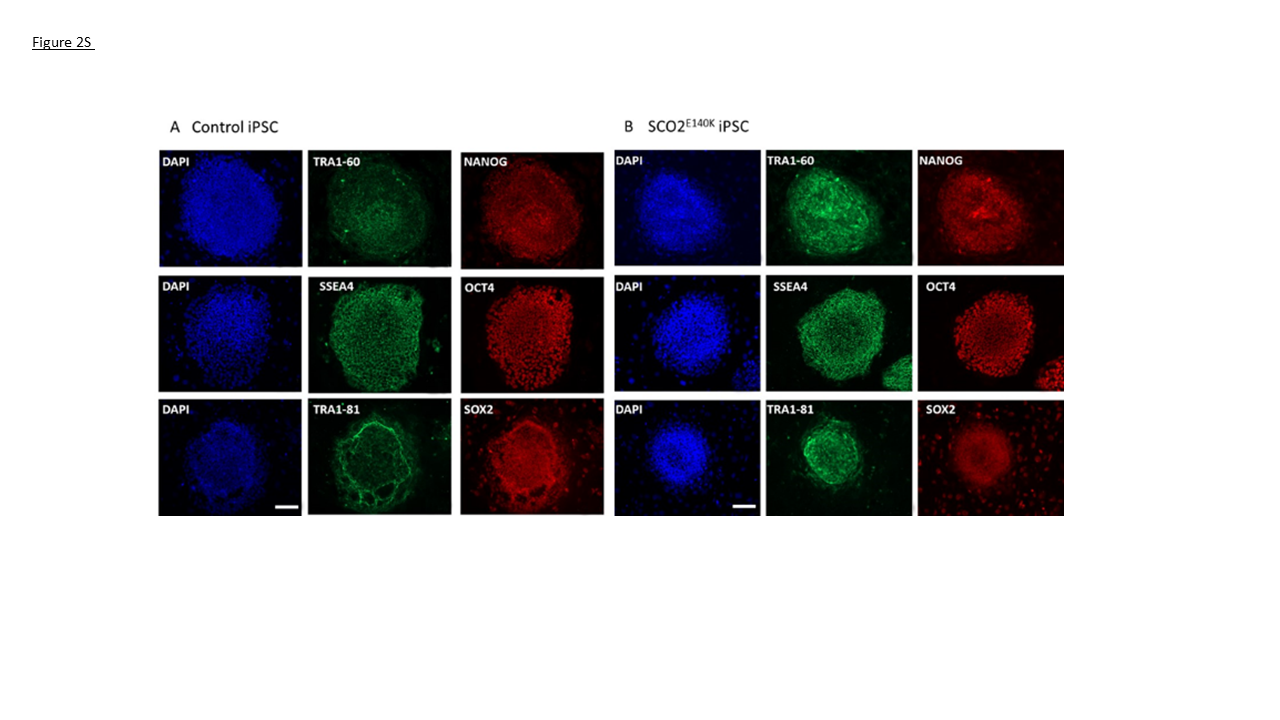

Supplement: Supplementary file 2 — Figure S2 Pluripotency of iPSC derived from the SCO2 patient and a healthy control. [file JCMM-22-913-s002.tif]

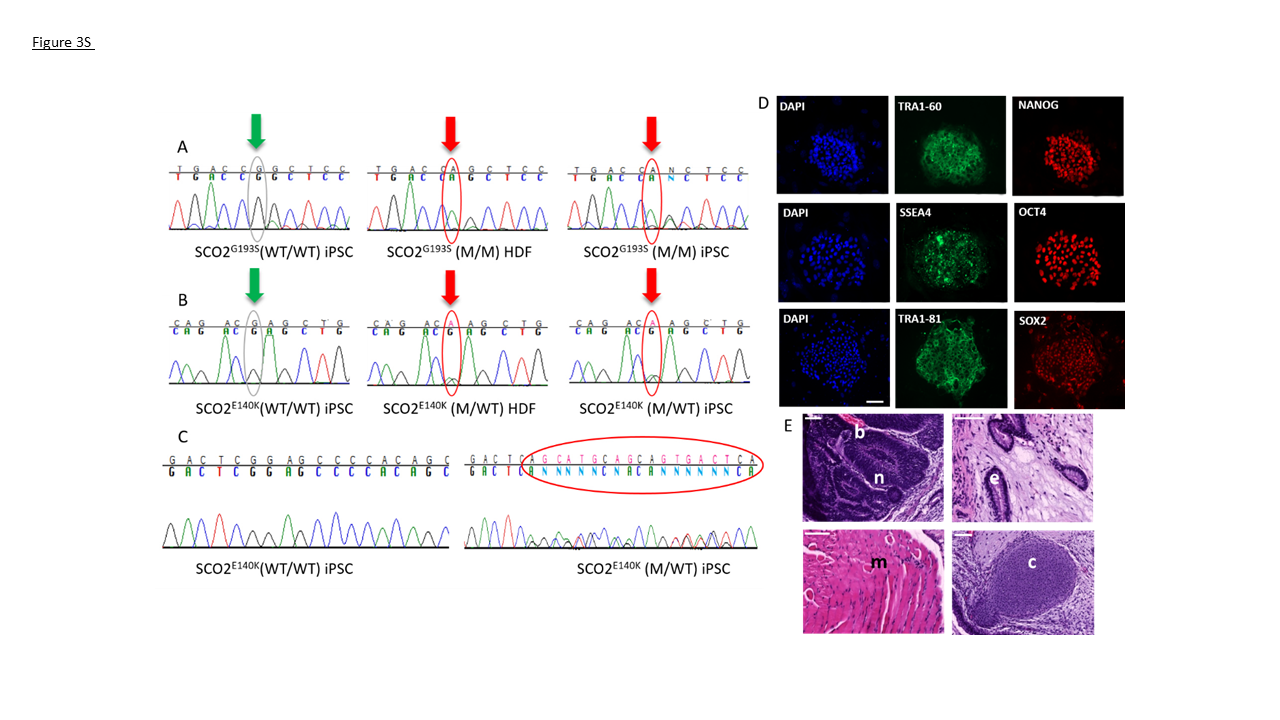

Supplement: Supplementary file 3 — Figure S3 Genetic, immunofluorescence and histological characterization of SCO2 iPSC. [file JCMM-22-913-s003.tif]

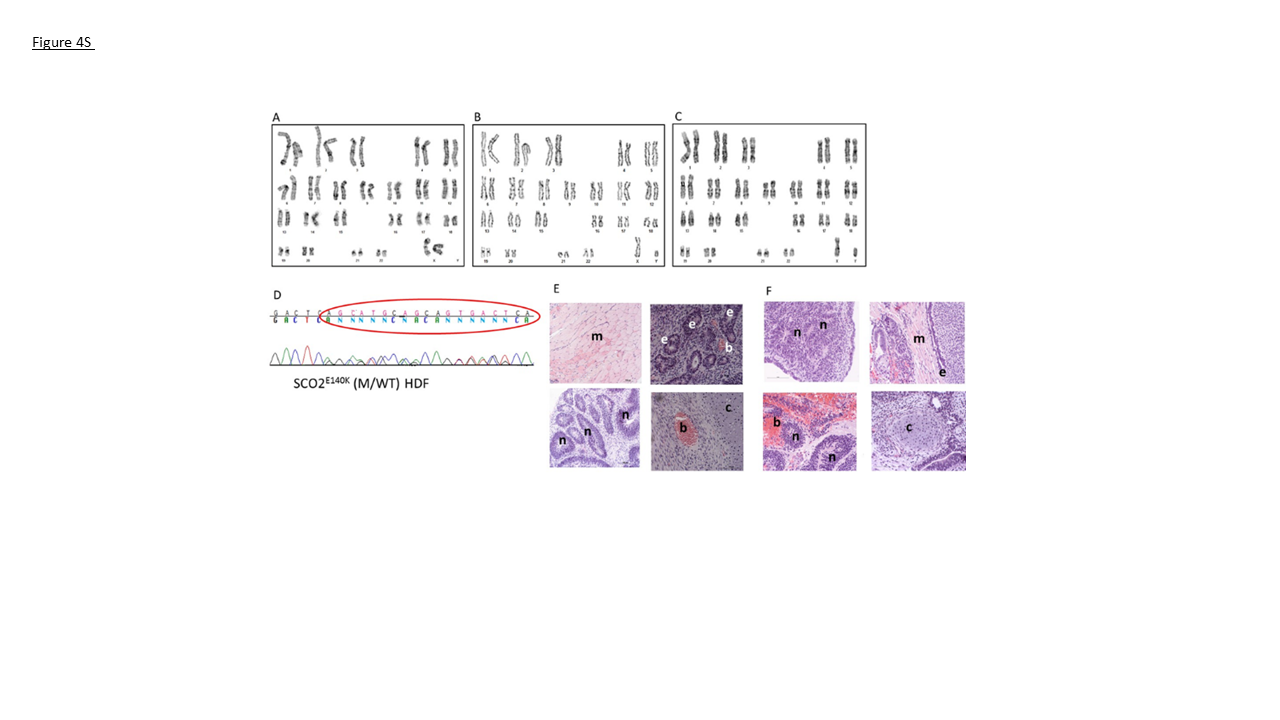

Supplement: Supplementary file 4 — Figure S4 Genetic and histological characterization of iPSC derived from SCO2 patients and a healthy control. [file JCMM-22-913-s004.tif]

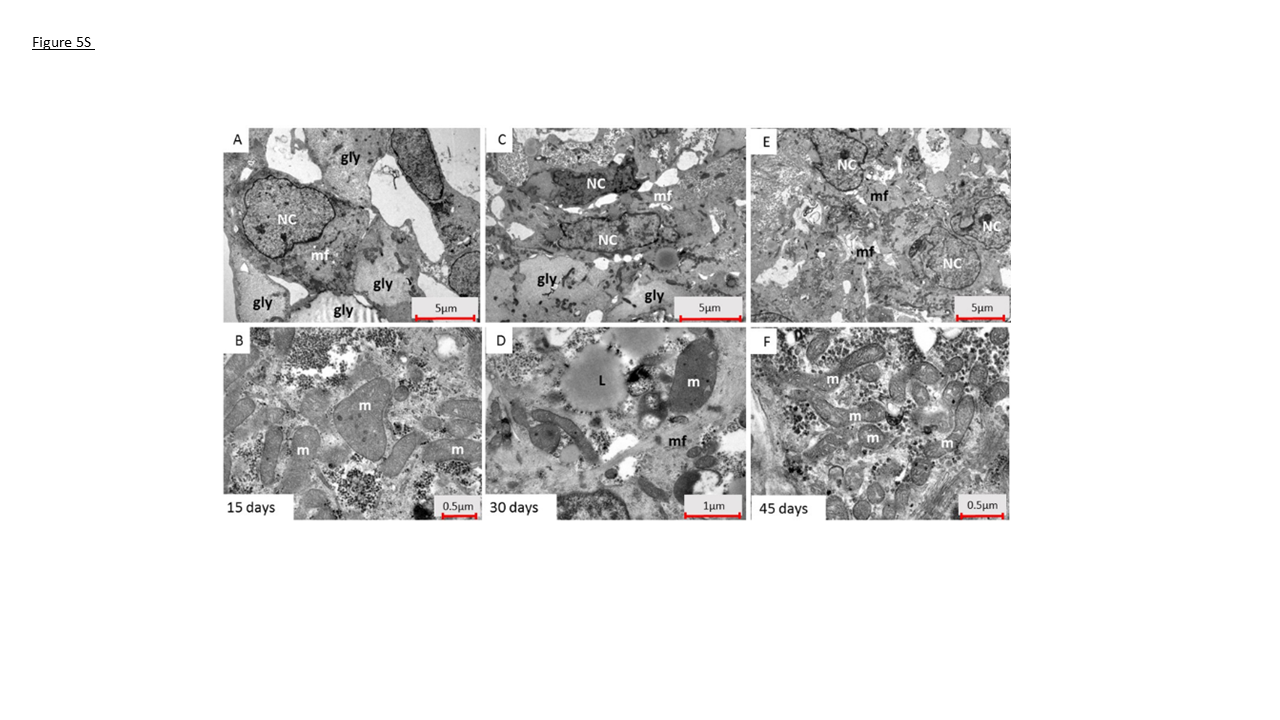

Supplement: Supplementary file 5 — Figure S5 Transmission electron microscopy (TEM) demonstrating ultrastructural abnormalities in SCO2 iPSC‐CM. [file JCMM-22-913-s005.tif]

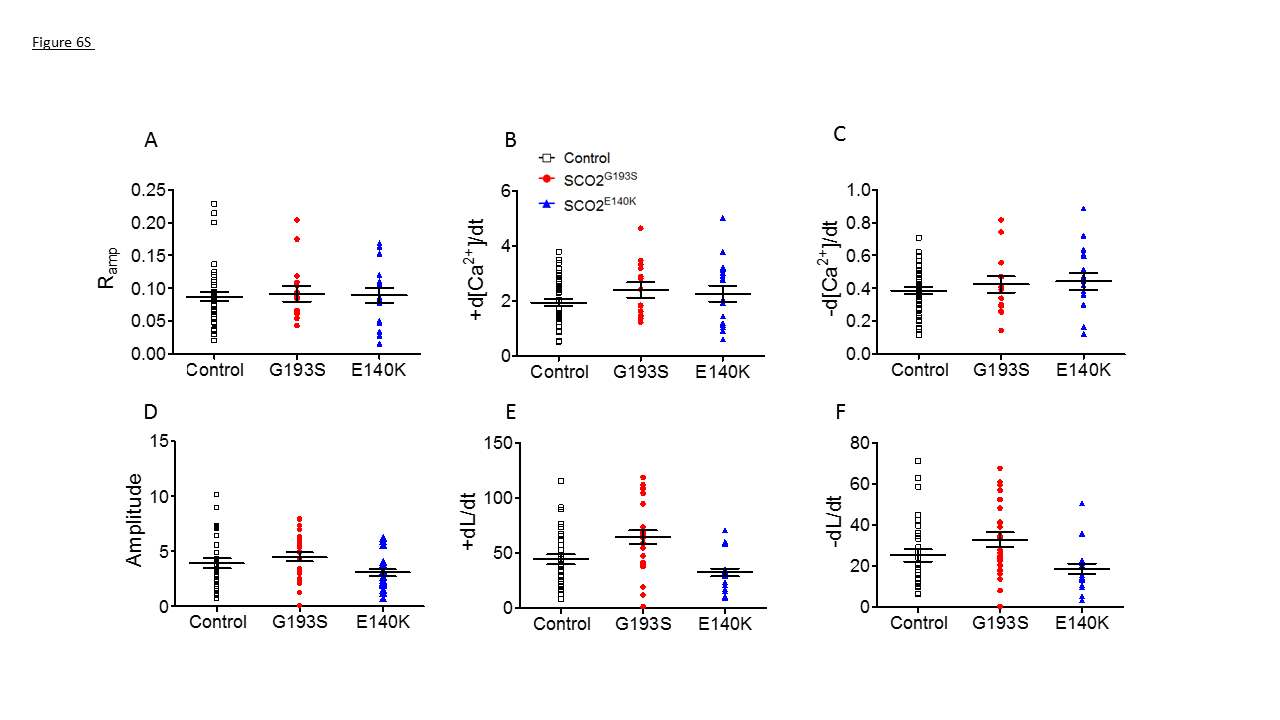

Supplement: Supplementary file 6 — Figure S6 Basal [Ca2+]i transients and contractions in control and SCO2 iPSC‐CMs. [file JCMM-22-913-s006.tif]

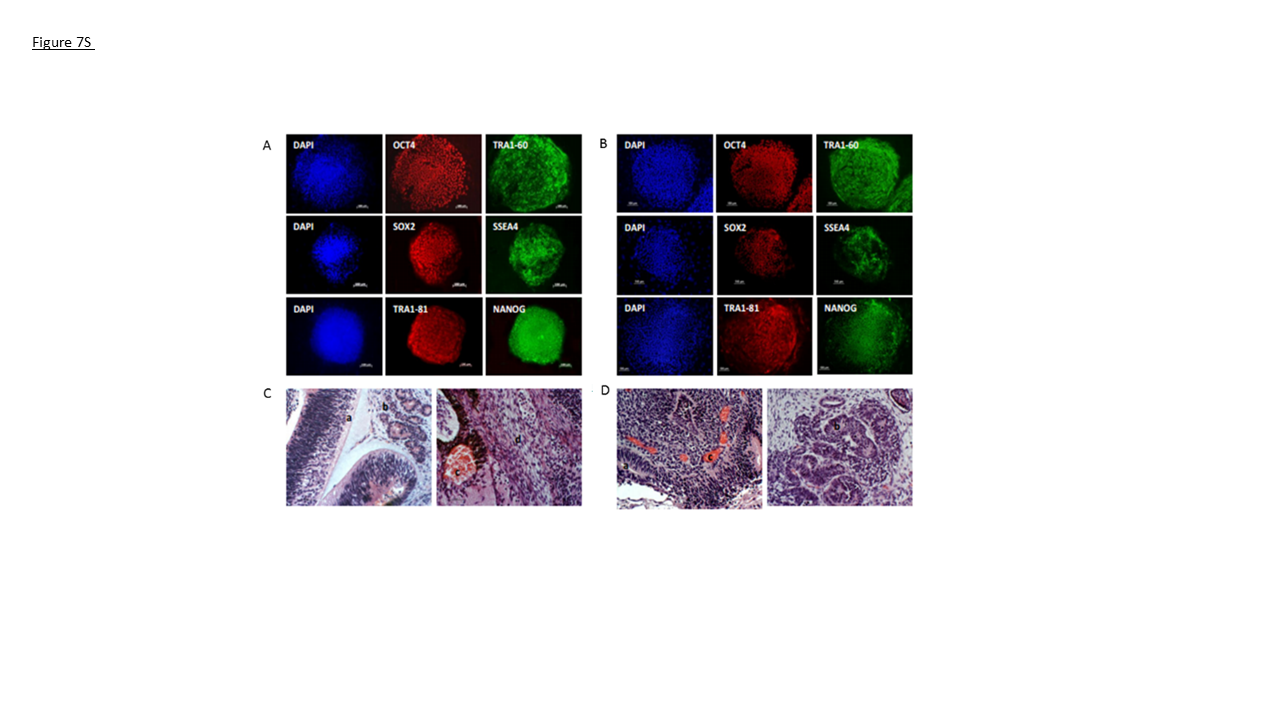

Supplement: Supplementary file 7 — Figure S7 Pluripotency of iPSC derived from human dermal fibroblasts and hair keratinocytes. [file JCMM-22-913-s007.tif]

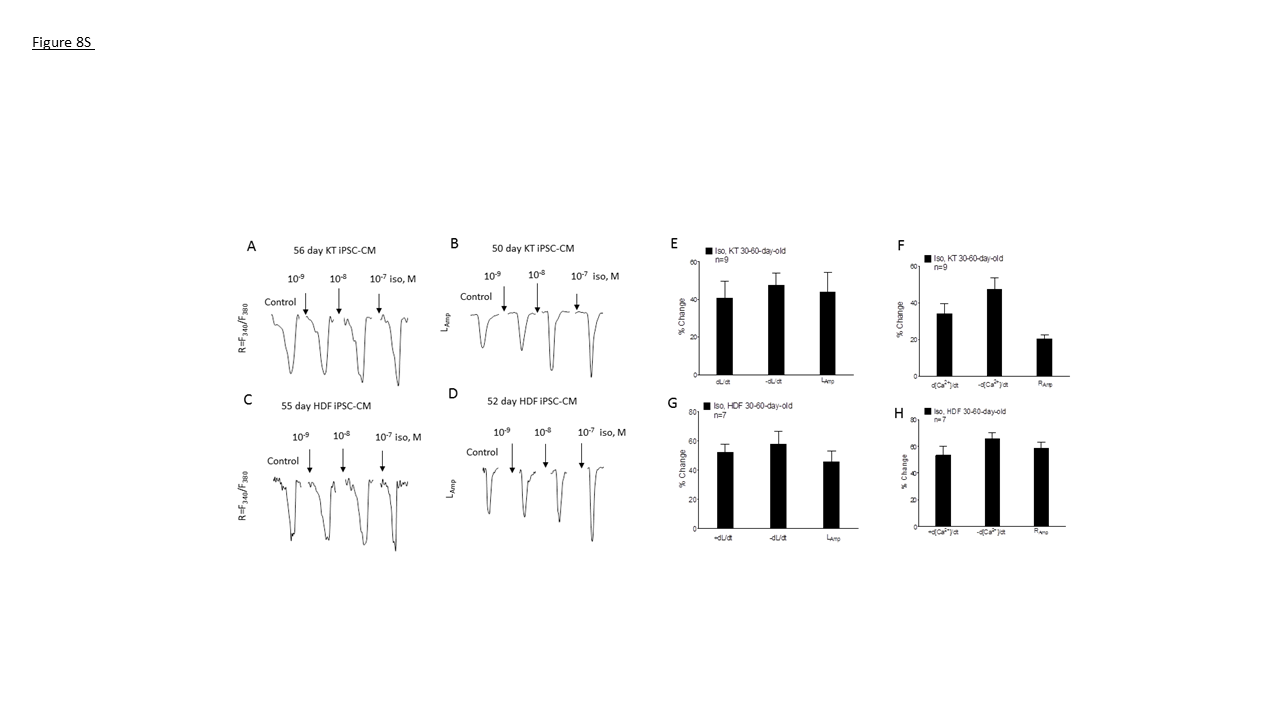

Supplement: Supplementary file 8 — Figure S8 Effect of isoproterenol on [Ca+2]i transients and contractions in KT and HDF iPSC‐CMs. [file JCMM-22-913-s008.tif]

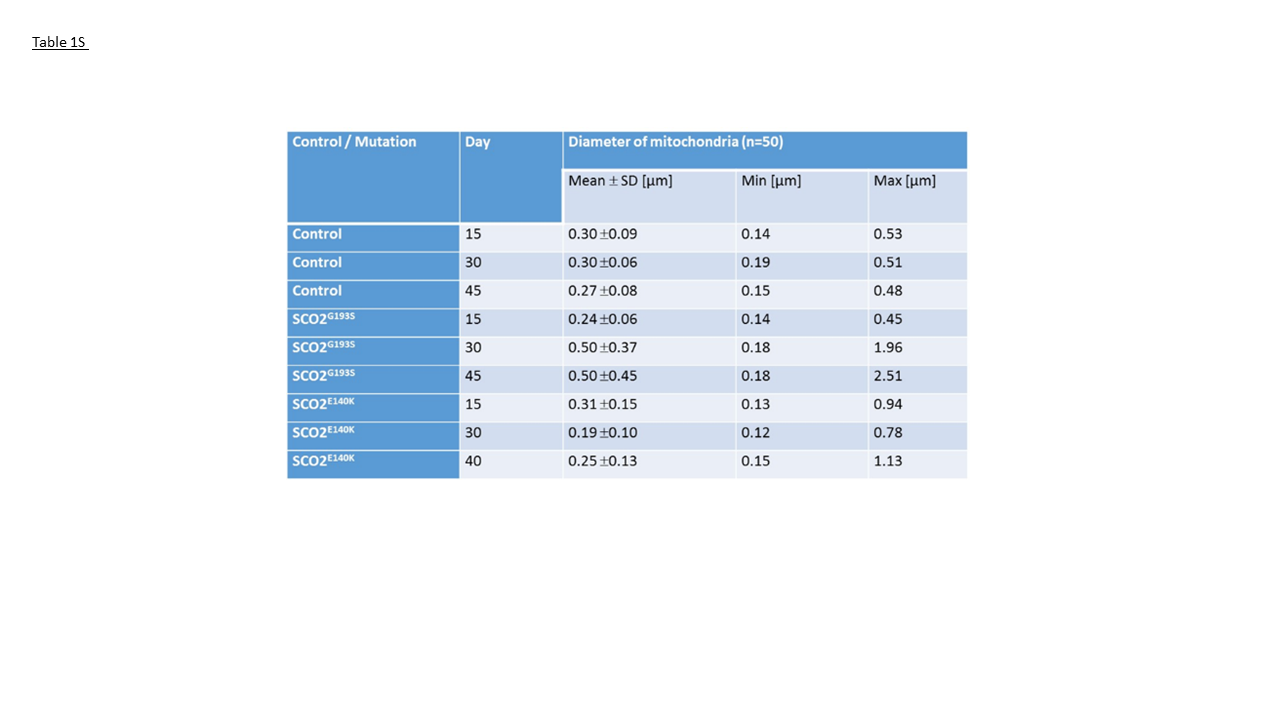

Supplement: Supplementary file 9 — Table S1. Mean diameter of mitochondria in control and SCO2‐mutated iPSC‐CMs. [file JCMM-22-913-s009.tif]
